# What Hong-Ou-Mandel interference says on two-photon frequency entanglement

**DOI:** 10.1038/s41598-017-07555-4

**Published:** 2017-08-03

**Authors:** Marco Barbieri, Emanuele Roccia, Luca Mancino, Marco Sbroscia, Ilaria Gianani, Fabio Sciarrino

**Affiliations:** 1 Dipartimento di Scienze, Università degli Studi Roma Tre, Via della Vasca Navale 84, 00146 Rome, Italy; 2 Dipartimento di Fisica, Sapienza Università di Roma, P.le Aldo Moro 5, 00185 Rome, Italy

## Abstract

Not much, in the end. Here we put forward some considerations on how Hong-Ou-Mandel interferometry provides signatures of frequency entanglement in the two-photon state produced by parametric down-conversion. We find that some quantitative information can be inferred in the limit of long-pulse pumping, while the short-pulse limit remains elusive.

## Introduction

As quantum technology are reaching beyond the proof-of-principle stage, the use of a reliable and robust way for encoding the information is becoming of paramount importance. The choice of encoding such information with internal degrees of freedom is preferable comparing to other well-established encodings (e.g. QKD with time-bins^1^) as it allows long-distance communications without deeply affecting the quantum state. In this context, by providing multiple modes, time-frequency correlations play a central role, finding use in many applications like QKD protocols^2–6^, high-rate fiber telecommunications^7^, and quantum ghost imaging^8^.

For these purposes, parametric down-conversion (PDC) constitutes one of the main enabling technologies for the generation of single photons and photon pairs. Due to the nature of the PDC process, the photon pairs produced by the scattering of pump photons on the nonlinear crystal, will exhibit correlations in frequency depending on the parameters defining the pump and the phase - matching function.

A key property of such two-photon states is their joint spectral amplitude, i.e. the joint probability for a spectral measurement of the two photons^9, 10^. While such a characterisation is generally sufficient to infer entanglement properties of the state, its experimental implementation is far from being trivial: it requires the implementation of frequency-resolved coincidence counting, with an unavoidable trade-off between spectral resolution and counting rate in any given frequency bin^11^. Achieving the resolution needed might be a hard task, and techniques have been developed for obtaining information about the quantum level starting from measurement in the stimulated classical regime, in order to overcome the trade off present in the purely quantum regime^12, 13^.

A more conventional approach would consist in performing quantum state tomography of the two-photon state. Since this requires the analysis of the frequency eigenbasis, as well as at least one rotated basis - i.e. one made of superposition states - to infer the complete state, we need fast frequency modulators to obtain superpositions. Encouraging results in this sense have been demonstrated^14–16^, however the modulation regime is not able to address the characteristic bandwidth of down-conversion with current technology. While nonlinear techniques could be used for frequency shearing^17^, their efficiency at the quantum level might prevent its application in practical cases. Alternative routes have considered the use of external references for reconstructing the spectral state of photons, presenting, however the difficulty of locking the apparatus to achieve phase stability^18, 19^.

A promising alternative is to look at interference in a Hong-Ou-Mandel (HOM) arrangement, which has already been demonstraded sensitive to spectral correlations^20^ and dispersion^21^: here, the two photons arrive within a short delay on a beam splitter via two different spatial inputs, and the coincidence counts at the outputs are recorded. As the delay is scanned, a characteristic interference figure is recorded, which, in the simplest case, has a dip in correspondence to zero delay. This figure has previously been demonstrated to depend explicitly on the spectral structure of the two-photon state, and that some of its feature can attest the presence of entanglement, although a quantitative estimate has not been obtained^22^. Recent studies have extended the usefulness of this technique for the reconstruction of the spectral wavefunction for the case of cw pumping^23, 24^. They effectively combine HOM interferometry with spectral shearing, obtained directly by tuning the two-photon source, hence avoiding extra nonlinear components. The technique - regardless some ambiguities of the reconstructed spectral phase - is powerful and can discriminate different wavefunctions generated in close, but distinct regimes, thanks to the redundancy obtained by multiple HOM interferogrammes for different shearing.

Here we address the question as to whether a single HOM interferogramme can provide quantitative estimation on the level of frequency entanglement of the two-photon state. We do not aim at providing a method for detecting entanglement unambiguously, but rather to understand whether a commonplace technique can be adopted by alignment operators for testing their source. We find that, to some extent, the HOM pattern can deliver some precious information on the spectral correlations within the state, proving a useful lower bound to the Schmidt number, counting the number of modes involved in the process.

## Results

We consider a two-photon state generated by a nonlinear process. Its spectral wavefunction is written as^9^:

1 $$|\psi \rangle =\int d{\omega }_{1}d{\omega }_{2}\,\alpha ({\omega }_{1}+{\omega }_{2})\varphi ({\omega }_{1},{\omega }_{2})\,{\hat{a}}_{{\omega }_{1}}^{\dagger }{\hat{a}}_{{\omega }_{2}}^{\dagger }\mathrm{|0}\rangle $$

where *α*(*ω*_1_ + *ω*_2_) is the pump spectral profile, and *ϕ*(*ω*_1_, *ω*_2_) is the material response function which depends on the phase matching; this latter generally takes the form of a simple sinc function, although this can be modified by the spatial pump profile, or by the presence of complex dispersion, as it is the case for four-wave mixing in resonators. The function ${\rm{\Phi }}({\omega }_{1},{\omega }_{2})=\alpha ({\omega }_{1}+{\omega }_{2})\phi ({\omega }_{1},{\omega }_{2})$ usually goes under the name of joint spectral amplitude (JSA), since it describes the probability amplitude of the detection of one photon at frequency *ω*_1_, and the other at *ω*_2_. Here we have assumed, as customary, a two-photon pure state at the output; this approximation is generally well-satisfied in down-conversion sources when spatial filtering is applied, such that the impact of space-time coupling is limited.

**Figure 1:**
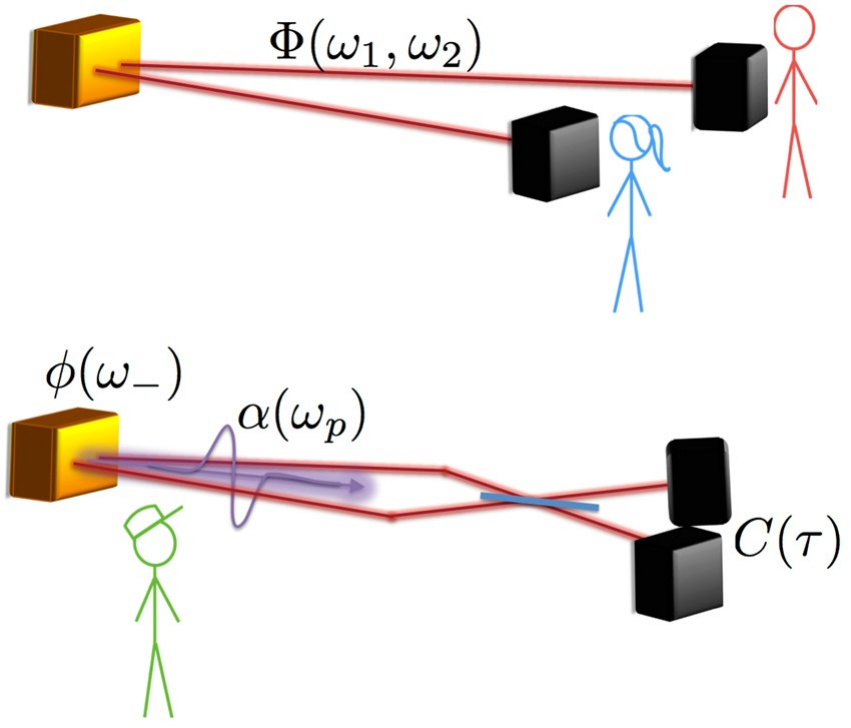
Conceptual layout of our analysis. In the common scenario, Alice and Bob need inferring entanglement in the joint spectral amplitude Φ(*ω*_1_, *ω*_2_) on the basis of local measurements. On the other hand, the handyman working on source development has access to information about the pump profile *α*(*ω*_*p*_), and can make the two modes interfere in a Hong-Ou-Mandel setup, thus inferring the approximated phase-matching function *ϕ*(*ω*___) from the coincidence pattern *C*(*τ*).

In HOM interferometry, the two photons are delivered onto a symmetric beamsplitter with a relative delay *τ* (see Fig. 1; we therefore require the nonlinear process to generate photon pairs close to the degenerate regime. The probability of a coincidence count at the outputs of the beam splitter depend on the delay as:

2 $$C(\tau )=\frac{1}{2}(1-{\rm{Re}}\,[\int d{\omega }_{1}d{\omega }_{2}\,{e}^{i({\omega }_{1}-{\omega }_{2})\tau }\,|\alpha ({\omega }_{1}+{\omega }_{2}{)|}^{2}{\varphi }^{\ast }({\omega }_{1},{\omega }_{2})\varphi ({\omega }_{2},{\omega }_{1})])$$

It is an established result that, whenever *C*(*τ*) surpasses the value 1/2, then the state exhibits entanglement in some degree of freedom; this ultimately descends from the fact that the modulation is sensitive to the anti-symmetric part of the wavefunction^22^.

The modulation *C*(*τ*) can be related to a decomposition of the JSA, that we can express as a convex combination of orthogonal separable functions *f*_*n*_ and *g*_*n*_^25^:

3 $${\rm{\Phi }}({\omega }_{1},{\omega }_{2})=\alpha ({\omega }_{1}+{\omega }_{2})\varphi ({\omega }_{1},{\omega }_{2})=\sum _{n}{\kappa }_{n}{f}_{n}({\omega }_{1}){g}_{n}({\omega }_{2}\mathrm{)}.$$

This represents the Schmidt decomposition of the JSA; a quantitative assessment of the level of entanglement is given by the cooperativity $K={({\sum }_{n}|{\kappa }_{n}{|}^{4})}^{-1}$, as an estimation of the number of modes involved in the decomposition^25^.

If we now insert the Schmidt decomposition in the HOM coincidence probability we get:

4 $$1-2C(\tau )={\rm{Re}}\,[\sum _{n,m}{\kappa }_{n}^{\ast }{\kappa }_{m}{F}_{n,m}(\tau ){F}_{m,n}{(\tau )}^{\ast }],$$

where we have introduced: ${F}_{n,m}(\tau )=\int d\omega \,{e}^{i\omega \tau }{f}_{n}^{\ast }(\omega ){g}_{m}(\omega )$, the convolution function between pairs of modes associated to individual photons. It is then clear that the HOM interference pattern reflects, in part, the correlation between the different possible pulse shapes available to the photons; however, the information can not be complete, since the pattern shape is only affected by the functional form JSA in the direction of *ω*_ = *ω*_1_ − *ω*_2_, the spectral phase also plays a limited role.

### Type II phase matching

**Figure 2:**
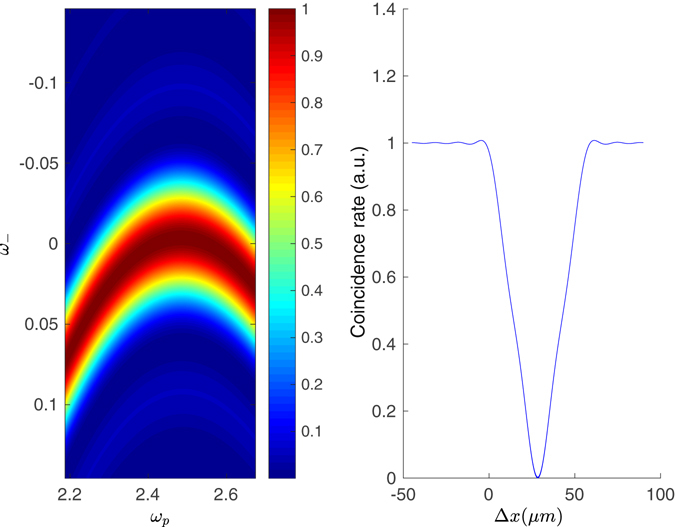
Left: the phase matching function $|\varphi {|}^{2}$, as a function of *ω*_*p*_ = *ω*_1_ + *ω*_2_, and *ω*_*_*_ = *ω*_1_ − *ω*_2_ for collinear Type II. Right: HOM coincidence pattern as a function of the delay Δ*x* = *cτ*. The count rate is normalised so to be 1 in the absence of interference, for long delays. Both panels refer to the case for a 2-mm *β*− barium borate (BBO) crystal, phase matched for 775 nm → 1550 nm conversion.

For the case of degenerate Type II phase matching the actual dependence on the sum frequency *ω*_1_ + *ω*_2_ modulates the phase matching function *ϕ*(*ω*_1_, *ω*_2_) over typical intervals which are larger than commonly-used pump bandwidths (see Fig. 2 for such an example), thus the dependence on *ω*_*p*_ = *ω*_1_ + *ω*_2_ can be neglected. This implies that, to a good approximation, one can write:

5 $${\rm{\Phi }}({\omega }_{1},{\omega }_{2})\simeq \alpha ({\omega }_{1}+{\omega }_{2})\varphi ({\omega }_{1}-{\omega }_{2}),$$

using the fact that around degeneracy the phase matching function is symmetric around zero in its variable *ω*_1_ − *ω*_2_. Our investigation aims at understanding the conditions in which this approximation effectively holds, hence extending the approach taken in refs 26, 27 for obtaining information on the spectral separability.

**Figure 3:**
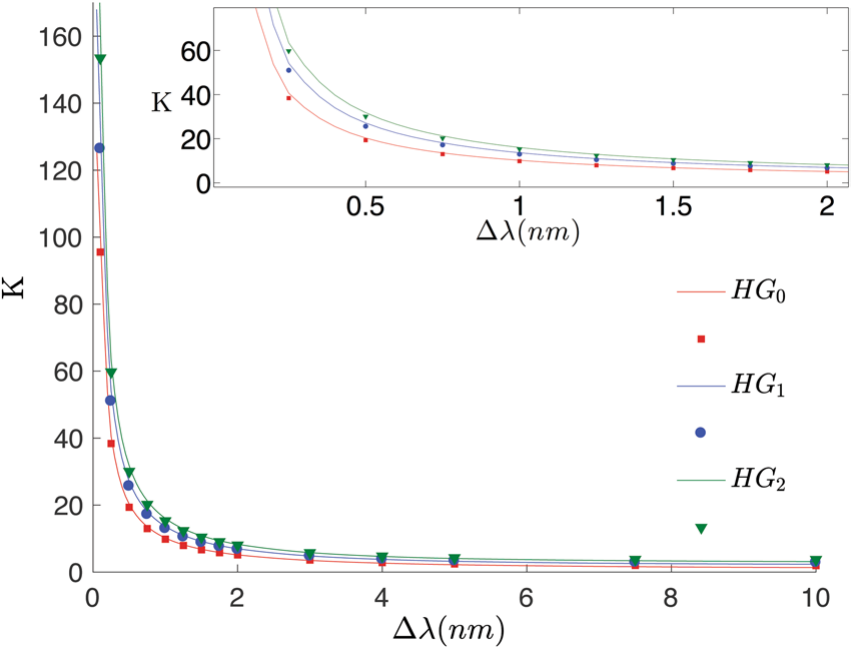
Spectral cooperativity *K*, estimated from the reduced model, Eq. (5), and from an exact calculation. We report the three cases of Hermite-Gauss spectra of order 0, 1, and 2, with spectral bandwidths Δ*λ*. The approximation holds to a satisfactory level, as shown in the inset which reports a close up of the whole curve. Notice how the number of modes generated increases with the order.

We start by a direct inspection of the HOM coincidence pattern in different pumping regimes, Fig. 2, corresponding to increasing bandwidths Δ*λ* of a Gaussian pulse $\alpha ({\omega }_{p})=exp(-\frac{({\omega }_{p}-{{\rm{\Omega }}}_{p})}{2{{\rm{\sigma }}}^{2}})$ - here Ω_*p*_ is the central pump frequency (in our example, it corresponds to *λ*_*p*_ = 775 *nm*), and σ is chosen so that Δ*λ* is the FWHM for the intensity spectrum. The shape of the HOM pattern is hardly affected when passing from quasi-monochromatic pumping, to the short-pulse conditions; the only variation is in the visibility of the pattern, which is hard to assess in an experiment with high precision, since it is affected by imperfections as the actual beam splitting ratio, or the presence of higher-order emission. Therefore, considering the pattern constant is convenient for any practical purpose; furthermore, its peculiar triangular shape can be taken as the result of the Fourier transform of a squared sinc function in (2):

6 $$\varphi ({\omega }_{-})=\text{sinc}({\tau }_{0}{\omega }_{-})$$

where the characteristic time *τ*_0_ can be estimated from the HOM pattern width. We now use the simple model 5 in order to estimate the cooperativity *K*, for the case of Hermite-Gauss pump profiles $\alpha ({{\rm{\omega }}}_{p})=$ $exp(-\frac{({\omega }_{p}-{{\rm{\Omega }}}_{p})}{2{{\rm{\sigma }}}^{2}}){H}_{n}(\frac{{\omega }_{p}-{{\rm{\Omega }}}_{p}}{{\rm{\sigma }}})$, (*H*_*n*_(*x*) being the *n*-th order Hermite polynomial). The results are shown in Fig. 3: the simple model is quite effective in providing a good estimation of the cooperativity, when compared to a detailed calculation of the JSA. Strictly speaking, as we are de facto insensitive to the phase structure, we are dealing with a way of finding a decomposition of the joint spectral *intensity*, although the impact of the phase is negligible in many practical cases. When only a few modes (of the order of 10 of less) are expected, the extracted value of *K* can be compared with the one inferred by a direct measurement of the unheralded second-order autocorrelation *g* ^(2)^(0) of the individual modes^10, 11^; a significative discrepancy would signal the presence of a non-trivial phase structure in the JSA, originating either because of the spectral phase of the pump, or of the material dispersion. The limitation in the number of modes arises from the need of effectively distinguishing *g* ^(2)^(0) from 1 in a photon counting experiment.

Our approximation seems to hold for a large interval of spectral widths, and also for moderately structured pump shapes. In order to obtain a guide for a judgement call, we can observe in Fig. 2 that variations to the phase-matching function with *ω*_*p*_ are relevant only on intervals comparable to the width of *ϕ* in the *ω*___ direction. Thus, we can consider, quite conservatively, our approximation to break when *K* ≲ 2, for pump pulses longer than the HOM pattern width: our approximation does not take into account all the characteristics of the crystal used, which may also be hard to access with sufficient precision. However these determine the behaviour of the cooperativity around its minimum *K* = 1, see Fig. 5. There, it could be hard to assess whether a value slightly above 1 does signal the genuine value of the cooperativity to the left of the minimum, or if we are slightly overestimating K to the right of the minimum, since the position of the minimum does depend on the actual crystal length and dispersion. Taking K = 2 as lower bound for this model is likely to be an overly-safe choice, while granting us the freedom of loosing the constraints on our knowledge of the crystal.

### Filtered type I phase matching

**Figure 4:**
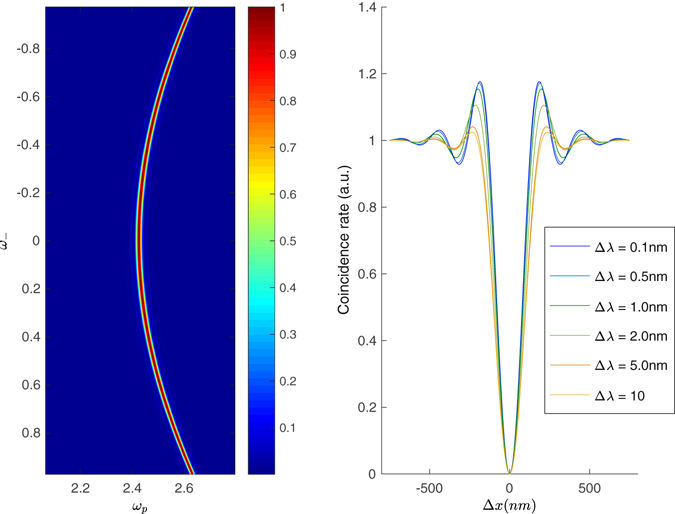
Left: the phase matching function $|\varphi {|}^{2}$, as a function of *ω*_*p*_ = *ω*_1_ + *ω*_2_, and *ω*_*_*_ = *ω*_1_ − *ω*_2_ for non-collinear type I. Right: HOM coincidence pattern as a function of the delay Δ*x* = *cτ*, for different pump bandwidths, measured as the FWHM of a Gaussian profile. The count rate is normalised so to be 1 in the absence of interference, for long delays. Both panels refer to the case for a 2-mm bismuth borate (BiBO) crystal, phase matched for 775 nm → 1550 nm conversion at an angle 9° with respect to the pump direction.

We can extend these considerations to type I phase matching, although it is important to remark that, in this case, filters will be necessary to reduce the large bandwidths (normally exceeding 50 nm) to more practical values. This is shown in Fig. 4, left panel, where the phase matching function $|\varphi ({\omega }_{1},{\omega }_{2}{)|}^{2}$ is reported for a typical non-collinear geometry. Notice that *ϕ* now extends over a much larger region than in the type-II case; such large bandwith is impractical in that any operation would be affected by the chromatism of the optical components. The spectral range is often limited by placing optical filters before the detectors.

In Fig. 4, right panel, shows the effect of using Gaussian pumps of increasing widths when performing a HOM interference experiment for given spectral filters - in our example, order-8 superGaussian filters with 10nm-FWHM that closely resemble a top-hat profile. For longer pump pulses, the interference figure retains the oscillations, characteristic of frequency entanglement, which are normally observed for cw pumping; shorter pump pulses result in the disappearance of such structures, as one would expect from the decreasing level of entanglement.

**Figure 5:**
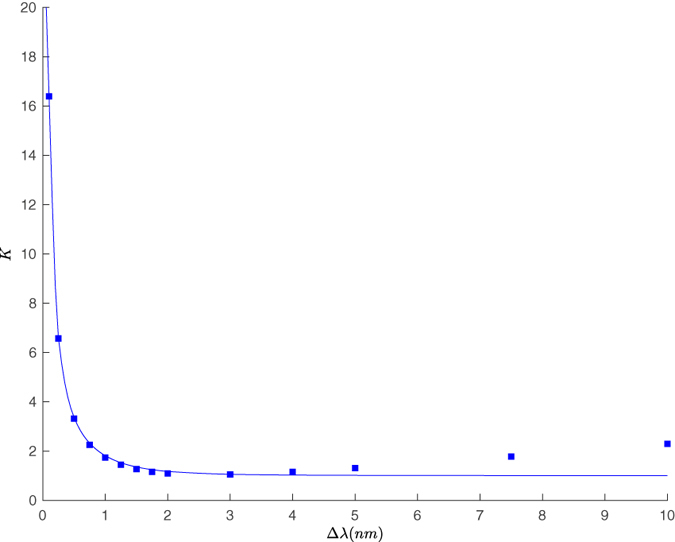
Spectral cooperativity *K*, estimated from the reduced model, Eq. (5), and from an exact calculation with spectral bandwidths Δ*λ*. The approximation holds to a satisfactory level.

We then apply the decomposition (5) in order to obtain an estimate of the JSA, and estimate the cooperativity *K*, with the results shown in Fig. 5; in this case, one can not rely on a simple *sinc* approximation, but the Fourier transform of the observed pattern has to be evaluated. It is recognised that proceeding with a direct transformation of the data would lead to instabilities due to noise and systematic errors, hence it is advisable to utilise in the experiment a suitable procedure for fitting the data. The approximation is satisfactory in the limit where the pump bandwidth is narrower than the relevant scale dictated by the filter FWHM. The width in the *ω*___-direction is still well-estimated by observing the HOM interference pattern, while the other direction can be grossly overestimated, due to the presence of the filters and to the limitations of the phase-matching bandwidth.

## Discussion

We have shown that it is possible to obtain informations on the correlations of time-frequency modes through commonplace HOM measurements. A single Hong-Ou-Mandel interferogramme can constitute a simple and easy resource for the characterization of time-frequency entanglement, without recurring to the experimentally demanding task of directly measuring the JSA. This procedure gives a satisfactory estimate with Type II phasematching and, due to filtering, only provides an upper bound in Type I when dealing with large-bandwidth pumps. As an application, the estimated cooperativity *K* can be compared to that estimated from the unheralded *g* ^(2)^(0) to diagnose the presence of effects such as phase correlations, or space-time coupling in phase matching^24^. Further, in the long-pulse regime, our method is expected to deliver an estimate for *K* even in conditions where *g* ^(2)^(0) ≃ 1; in this regime, there is also an experimental advantage in decoupling the two directions along *ω*_*p*_ and *ω*___ in a way that a detector with the suitable resolution can be used for each of them.
